# Differential Gene Expression of Cardiac Ion Channels in Human Dilated Cardiomyopathy

**DOI:** 10.1371/journal.pone.0079792

**Published:** 2013-12-05

**Authors:** Maria Micaela Molina-Navarro, Esther Roselló-Lletí, Ana Ortega, Estefanía Tarazón, Manuel Otero, Luis Martínez-Dolz, Francisca Lago, José Ramón González-Juanatey, Francisco España, Pablo García-Pavía, José Anastasio Montero, Manuel Portolés, Miguel Rivera

**Affiliations:** 1 Cardiocirculatory Unit, Health Research Institute Hospital La Fe, Valencia, Spain; 2 Cellular and Molecular Cardiology Research Unit, Department of Cardiology and Institute of Biomedical Research, University Clinical Hospital, Santiago de Compostela, Spain; 3 Cardiovascular Surgery Service, University Hospital La Fe, Valencia, Spain; 4 Hemostasis, Thrombosis, Atherosclerosis and Vascular Biology Unit, Health Research Institute Hospital La Fe, Valencia, Spain; 5 Cardiology Service, University Hospital Puerta del Hierro, Madrid, Spain; 6 Cell Biology and Pathology Unit, Health Research Institute Hospital La Fe, Valencia, Spain; University of Buenos Aires, Faculty of Medicine. Cardiovascular Pathophysiology Institute., Argentina

## Abstract

**Background:**

Dilated cardiomyopathy (DCM) is characterized by idiopathic dilation and systolic contractile dysfunction of the cardiac chambers. The present work aimed to study the alterations in gene expression of ion channels involved in cardiomyocyte function.

**Methods and Results:**

Microarray profiling using the Affymetrix Human Gene® 1.0 ST array was performed using 17 RNA samples, 12 from DCM patients undergoing cardiac transplantation and 5 control donors (CNT). The analysis focused on 7 cardiac ion channel genes, since this category has not been previously studied in human DCM. *SCN2B* was upregulated, while *KCNJ5*, *KCNJ8*, *CLIC2*, *CLCN3*, *CACNB2*, and *CACNA1C* were downregulated. The RT-qPCR (21 DCM and 8 CNT samples) validated the gene expression of *SCN2B* (p < 0.0001), *KCNJ5* (p < 0.05), *KCNJ8* (p < 0.05), *CLIC2* (p < 0.05), and *CACNB2* (p < 0.05). Furthermore, we performed an IPA analysis and we found a functional relationship between the different ion channels studied in this work.

**Conclusion:**

This study shows a differential expression of ion channel genes involved in cardiac contraction in DCM that might partly underlie the changes in left ventricular function observed in these patients. These results could be the basis for new genetic therapeutic approaches.

## Introduction

Dilated cardiomyopathy (DCM) is one of the most frequent diseases that cause heart failure (HF) [1]. DCM is characterized by idiopathic dilation and systolic contractile dysfunction, with an increase in ventricular mass and volume and wall thickness [2]. Ion channel disruptions have been described as contributory to the development of DCM [3]. Nevertheless, there are not studies analyzing the mechanisms involved in cardiac contraction dysfunction at the ion channel gene expression level.

Cardiac muscle contraction produced by the initiation of action potentials (AP) in cardiomyocytes has an important role in the pathogenesis of the disease. Cardiac ion channels are responsible for ion currents that determine and influence the cardiac AP in different parts of the human heart [4]. Furthermore, cardiomyocytes are highly differentiated cells that specialize in excitation-contraction (EC) coupling, and have well-developed mechanical and electrical properties. The sarcomere is the functional unit in the contraction process that spans the area between the Z lines. It is made of three types of filaments: thin (actin), thick (myosin), and elastic (titin or connectin) [5]. Ca^2+^ ions play an important role through binding directly to sarcomeric proteins allowing the initiation of the myocyte contraction [6,7].

The major ion channels involved in both the depolarization and repolarization of muscle cells are implicated in sodium, potassium, calcium, and chloride ion fluxes [8,9]. A common structure exists in all ion channels, including a transmembrane subunit α that forms the ion-conducting pore, and a variable number of associated subunits that are responsible for the regulation of channel expression and gating [10-12].

Establishing the alterations in gene expression is a proper manner to elucidate the causes or putative treatments of many diseases. We used high-throughput whole-genome microarray as well as the database for annotation, visualization and integrated discovery (DAVID) analysis tool to determine the biological and functional categories of the obtained gene list.

Since low contraction is one of the causes of poor prognosis in patients with DCM, we hypothesized that patients with DCM may show changes in the expression of genes related to cardiac contraction, such as genes encoding ion channels. Therefore, the aim of the study was to evaluate for the first time the differential gene expression of cardiac ion channels in DCM patients compared to control subjects. 

## Methods

### Ethics statement

The project was approved by the Ethics Committee of Hospital La Fe, Valencia, and all participants gave their written, informed consent. The study was conducted in accordance with the guidelines of the Declaration of Helsinki [13].

### Source of tissue

Experiments were performed with left ventricular (LV) samples from explanted human hearts from patients with DCM undergoing cardiac transplantation. Clinical history, hemodynamic study, ECG, and Doppler echocardiography data were available from all of these patients. Non-ischemic DCM was diagnosed when patients had LV systolic dysfunction (EF <40%) with a dilated non-hypertrophic left ventricle (LVDD >55 mm) on echocardiography. Moreover, patients did not show existence of primary valvular disease and familial DCM. All patients were functionally classified according to the New York Heart Association (NYHA) criteria and they were receiving medical treatment following the guidelines of the European Society of Cardiology [14].

Non-diseased donor hearts were used as control (CNT) samples. The hearts were initially considered for transplantation, but were subsequently deemed unsuitable for transplantation either because of blood type or size incompatibility. The cause of death was cerebrovascular or motor vehicle accident. All donors had normal LV function and had no history of myocardial disease or active infection at the time of transplantation. 

Transmural samples were taken from near the apex of the left ventricle and stored at 4°C for a maximum of 6 h from the time of coronary circulation loss. Samples were stored at -80°C until the RNA and protein extractions were performed.

Of 29 heart samples, 17 were used in the microarray profiling (DCM, n = 12; and CNT n = 5). The 29 total heart samples were used in the validation by RT-qPCR to improve the numerical base with a higher number of patients and control subjects (DCM, n = 21; and CNT, n = 8).

### Total RNA isolation

RNA was extracted using a Qiagen RNeasy Fibrous Tissue Mini kit following the manufacturer’s instructions (Qiagen Iberia SL, Spain). The concentration of the obtained RNA was assessed using a NanoDrop 2000 spectrophotometer and the quality was determined using a microfluidic-based platform (2100 Bioanalyzer, Agilent Technologies, Spain SL).

### Microarray analysis

cDNA synthesis was carried out using the WT Expression Sense Target Protocol (Ambion, Life Technologies, Carlsbad, CA, USA), and genome-wide gene expression was determined using Affymetrix Human Gene® 1.0 ST arrays (Affymetrix, Santa Clara, CA, USA) according to the manufacturer’s instructions. Array hybridization, washing, and scanning were performed using the Gene Chip Scanner 7G System platform (Affymetrix, Santa Clara, CA, USA). The GeneChip® Command Console software was used for initial image processing. Affymetrix Expression Console^TM^ software provided quality control and a probe set summarization to attain gene-level signal data (Affymetrix, Santa Clara, CA, USA). The Partek® Genomics Suite^TM^ (Partek Inc., Saint Louis, MO, USA) software was used for background correction, normalization, probe summarization and statistical comparison (ANOVA) of expression profiles between the pathological group and the control group using the RMA algorithm. Genes were considered significantly different with a p-value <0.001 and a fold change of 1.3. All quantitative results are available at the NIH GEO database (GEO #GSE42955). DAVID programme was used to classify genes functionally associated with the aim to explore alterations in these functional categories following the published protocol for DAVID [15].

### Real-time quantitative PCR analysis

We performed a quantitative real-time polymerase chain reaction (RT-qPCR) on frozen heart specimens from pathological and control subjects. Reverse transcription was carried out using 1 µg total RNA and Superscript III (Invitrogen Ltd, UK) according to the manufacturer’s protocol. The resulting cDNA was used as the template for RT-qPCR in a high-throughput thermocycler (ViiA^TM^ 7 Real-Time PCR System, Applied Biosystems, Foster City, CA, USA) according to the manufacturer’s instructions. The following TaqMan® probes were used: *SCN2B* (*Hs00394952_m1*), *KCNJ5* (*Hs00942581_m1*)*, KCNJ8* (*Hs00958961_m1*), *CLIC2* (*Hs01574555_m1*), *CLCN3* (*Hs00923161_m1*), *CACNB2* (*Hs00167861_m1*), and *CACNA1C* (*Hs00167681_m1*). Quantification of gene expression was normalized to *GAPDH* (*Hs99999905_m1*), *PGK1* (*Hs99999906_m1*), and *TFRC* (*Hs00951083_m1*) as endogenous controls. And as a positive control of the RT-qPCR experiment, we analyzed the gene expression level of the genes *KCND3* (*Hs00542597_m1*) and *ATPA2A* (*Hs00544877_m1*) which have shown a downregulation in human HF [16-20] (Figure S1). Relative gene expression levels were calculated using the 2^-ΔΔCT^ method [21].

### Homogenization of samples and protein determination

Thirty milligrams of frozen left ventricles were transferred into Lysing Matrix D tubes designed for use with the FastPrep-24 homogenizer (MP Biomedicals, USA) in total protein extraction buffer (2% SDS, 10 mM EDTA, 6 mM Tris-HCl, pH 7.4) with protease inhibitors (25 µg/mL aprotinin and 10 µg/mL leupeptin). The homogenates were centrifuged and the supernatants aliquoted. The protein content of aliquots was determined using Peterson’s modification of the micro Lowry method, using bovine serum albumin (BSA) as a standard [22].

### Gel electrophoresis and Western blot analysis

Protein samples for detection of SCN2B and KCNJ5 were separated by Bis-Tris electrophoresis on 4–12% polyacrylamide gels under reducing conditions. After electrophoresis, the proteins were transferred from the gel to a PVDF membrane using the iBlot Dry Blotting System (Invitrogen Ltd, UK) for Western blot analyses. The membrane was blocked all night at 4°C with 1% BSA in Tris-buffer solution containing 0.05% Tween 20 and then for 2 h with a primary antibody in the same buffer. The primary detection antibodies used were anti-SCN2B rabbit polyclonal antibody (1:200), and anti-KCNJ5 rabbit polyclonal antibody (1:500). Anti-GAPDH mouse monoclonal antibody (1:1000) was used as a loading control. All antibodies used were from Abcam (Cambridge, UK).

Bands were visualized using an acid phosphatase-conjugated secondary antibody and nitro blue tetrazolium/5-bromo-4-chloro-3-indolyl phosphate (NBT/BCIP, Sigma) substrate system. Finally, the bands were digitalized using an image analyzer (DNR Bio-Imagining Systems, Israel) and quantified by the GelQuant Pro (v12.2) program.

### Pathway analysis

Ingenuity Pathway Analysis (IPA) software (Ingenuity® Systems, www.ingenuity.com) was used to detect the biological pathways of the differentially expressed ion channel genes using the human Refseq IDs as input. Biological groups that were significantly associated with the genes of interest (p < 0.05) were identified. 

### Statistics

Data are presented as the mean ± standard deviation (SD). The Kolmogorov–Smirnov test was used to analyze the normal distribution of the variables. Comparisons between 2 groups were performed using Student’s *t*-test, and Pearson’s correlation coefficient was calculated to analyze the association between variables. Analyses were considered significant when p < 0.05. All statistical analyses were performed using SPSS software v. 20 for Windows (IBM SPSS Inc., Chicago, IL, USA).

## Results

### Clinical characteristics of patients

Samples from 12 explanted human hearts from patients diagnosed with DCM undergoing cardiac transplantation and 5 non-diseased donor hearts as CNT samples were used in the microarray profiling analysis. All the patients were men with a mean age of 48 ± 9 years, a mean NYHA functional classification of III-IV, and previously diagnosed significant comorbidities, including hypertension and hypercholesterolemia. Table 1 shows the clinical characteristics of patients according to the etiology of DCM. 

**Table 1:** Clinical characteristics of patients with dilated cardiomyopathy.

	**Microarray experiment**	**RT-qPCR**
	**DCM (n = 12)**	**DCM (n = 21)**
**Age (years)**	48 ± 9	49 ± 14
**Gender male (%)**	100	96
**NYHA class**	3.5 ± 0.4	3.2 ± 0.4
**BMI (kg/m^2^)**	26 ± 4	27 ± 7
**Hemoglobin (mg/mL)**	13 ± 2	13 ± 2
**Hematocrit (%)**	40 ± 6	39 ± 6
**Total cholesterol (mg/dL)**	158 ± 45	136 ± 41
**EF (%)**	20 ± 6	22 ± 7
**FS (%)**	10 ± 3	12 ± 4
**LVESD (mm)**	71 ± 10	62 ± 10
**LVEDD (mm)**	79 ± 9	71 ± 10
**LV mass index (g/cm^2^)**	206 ± 52	191 ± 53

DCM, dilated cardiomyopathy; NYHA, New York Heart Association; BMI, body mass index; EF, ejection fraction; FS, fractional shortening; LVESD, left ventricular end-systolic diameter; LVEDD, left ventricular end-diastolic diameter; LV mass index, left ventricular mass index.

We increased the sample size to improve the analysis up to 21 DCM and 8 CNT hearts for the RT-PCR validation assay, the clinical characteristics of these DCM patients are also shown in Table 1.

The control group used for the microarray profiling was comprised of 80 % men with 55 ± 3 years. And in the increased sample size used for the validation process, 60% were men with 45 ± 14 years.

### Gene expression profiling

**Figure 1:**
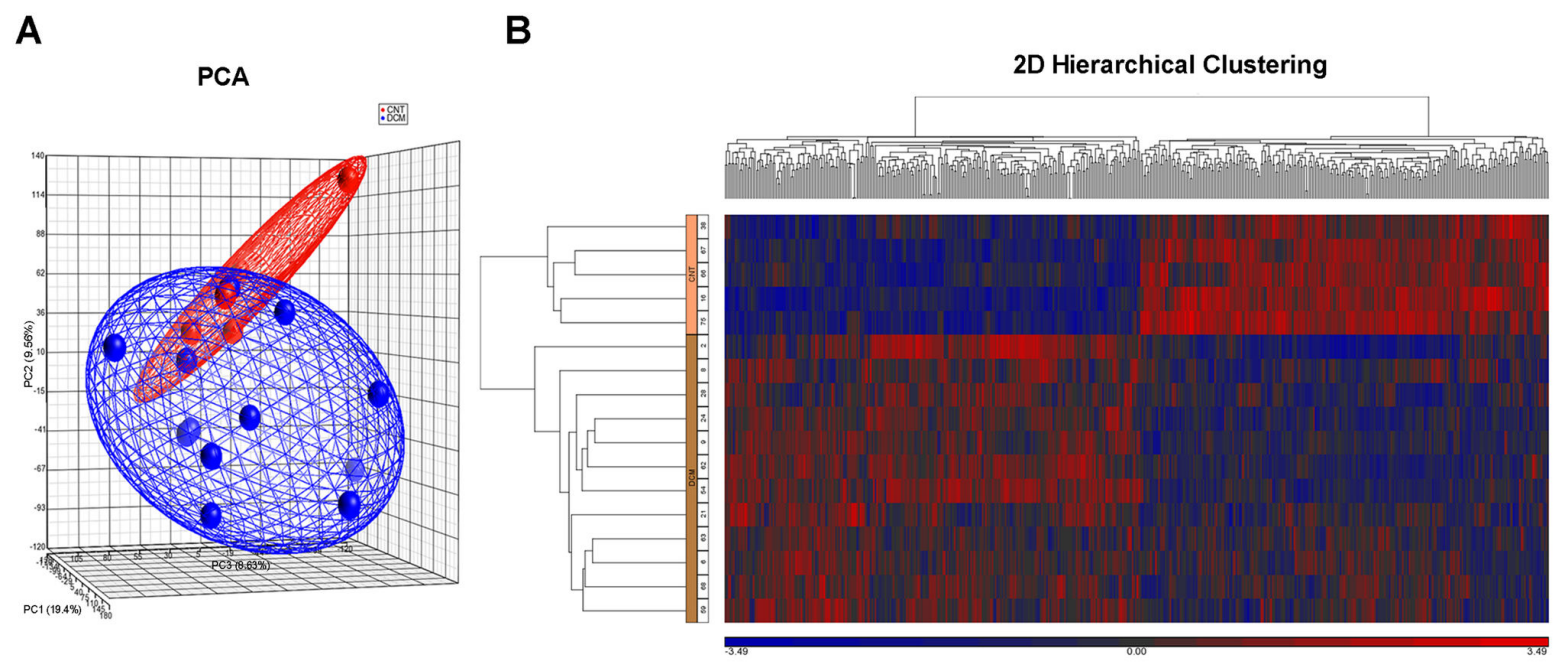
A. PCA analysis of the expression data clusters patients and controls into their respective groups. B. 2D Hierarchical clustering (p-value <0.01 and FC ≥1.3) reveals the existence of 2 sample groups, DCM and CNT, clearly separated.

A gene expression microarray was performed to determine gene expression differences between the DCM and CNT groups. The results of the microarray experiment are shown in Table S1. A quality control for hybridization was carried out before the statistical analysis obtained using Partek Genomics Suite. Multivariate analysis, in the form of principal component analysis (PCA) was used to compare the expression profile of the sample groups based on their comprehensive expression profiles. The score plot obtained showed that 21.2 % of the differences among the sample groups could be explained by PCA component 1, 10.4 % by PCA component 2, while PCA component 3 explained 9.95 % of the differences (Figure 1A). A hierarchical clustering in both dimensions (samples and genes) showed clear differentiation between the pathological and control groups without any degree of overlap (Figure 1B). 

The comparison of DCM patients with the CNT group showed 503 genes differentially expressed (p-value <0.001 and fold change >1.3), of which 201 were upregulated and 302 were downregulated (Table S1).

Among these differentially expressed genes, 13 belonged to the cardiac voltage-gated ion channel activity functional category according to the DAVID programme (Table S2). These genes are responsible for ion trafficking involved in cardiac contraction, an important process compromised in DCM. As this functional category has yet to be characterized in DCM, we focused on 7 of these ion channels (*SCN2B*, *KCNJ5*, *KCNJ8*, *CLIC2*, *CLCN3*, *CACNB2*, and *CACNA1C*) in this study, based on the described relationship of these channels with the contraction process (Table 2).

**Table 2:** Cardiac ion channel genes with differential expression in microarray profiling and selected for validation.

**Gene**	**Description**	**Fold Change**	**p-value**
***SCN2B***	Sodium channel subunit beta-2	2.03	5.20 x 10^-6^
***KCNJ5***	G protein-activated inward rectifier potassium channel 4	-1.95	6.44 x 10^-4^
***KCNJ8***	ATP-sensitive inward rectifier potassium channel 8	-1.44	6.72 x 10^-3^
***CLIC2***	Chloride intracellular channel protein 2	-1.77	4.87 x 10^-4^
***CLCN3***	H(+)/Cl(-) exchange transporter 3	-1.39	7.82 x 10^-3^
***CACNB2***	Voltage-dependent L-type calcium channel subunit beta-2	-1.51	1.92 x 10^-3^
***CACNA 1C***	Voltage-dependent L-type calcium channel subunit alpha-1C	-1.37	9.95 x 10^-3^

### Real-time quantitative PCR analysis

**Figure 2:**
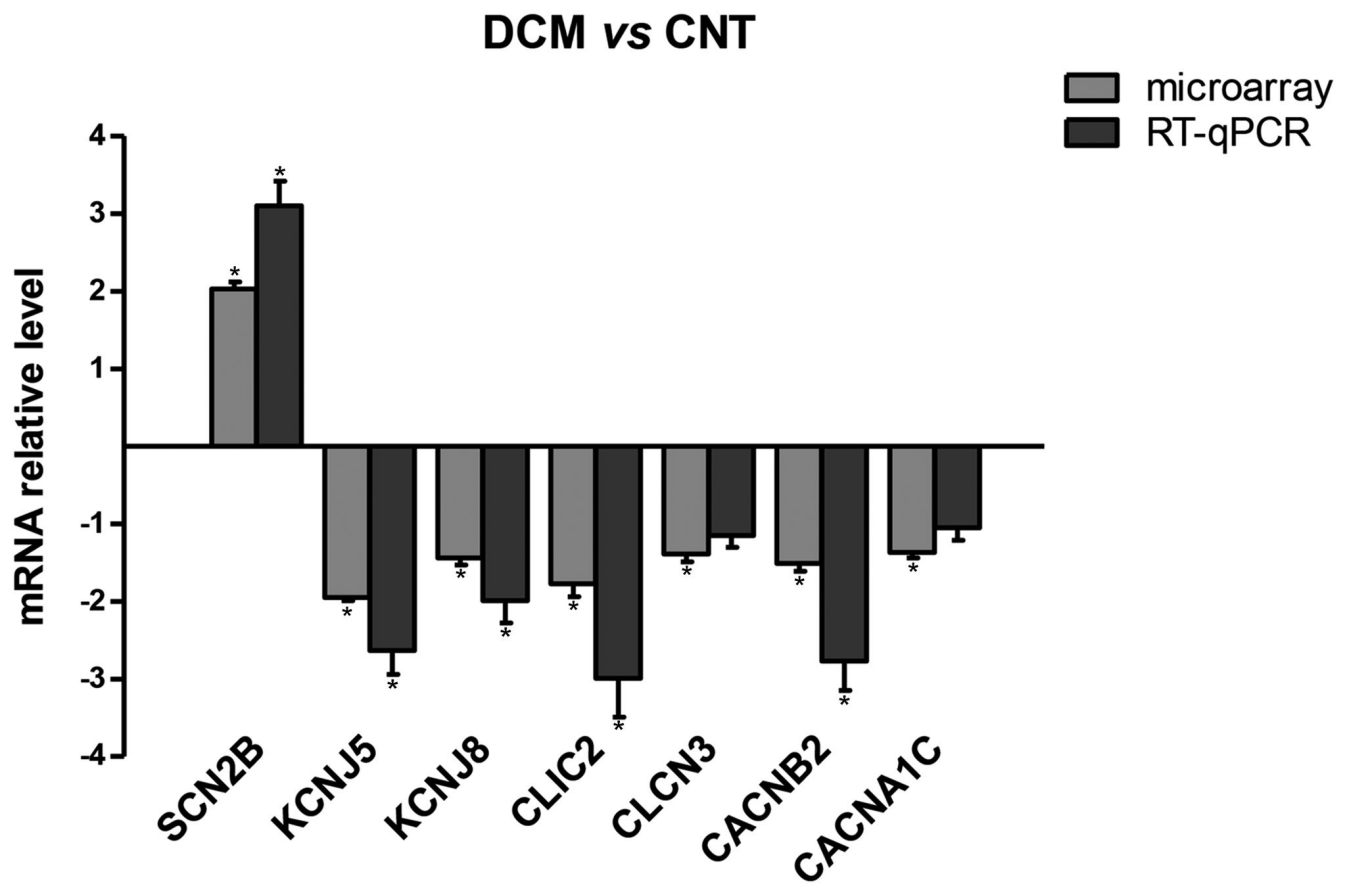
Verification of microarray data by RT-qPCR. The graph depicts the values obtained in microarrays and relative mRNA levels obtained using RT-qPCR normalized to the mRNA expression of 3 housekeeping genes (GAPDH, PGK1, and TFRC), respectively. The error bar represents the standard error of the mean (SEM) for DCM (n =12) and CNT (n = 5) samples in microarray data, and for DCM (n =21) and CNT (n = 8) samples in RT-qPCR data. * p < 0.05 *vs*. CNT.

RT-qPCR was performed to validate the results obtained in the microarray profiling experiment using both the same samples used in the microarray and new samples for a total of 21 DCM and 8 CNT subjects. It was shown that *SCN2B* was upregulated, while *KCNJ5*, *KCNJ8*, *CLIC2*, and *CACNB2* were downregulated in DCM compared to CNT (Figure 2), confirming the microarray results with regard to fold change and significance. However, the expression of *CACNA1C* and *CLCN3* was not significantly altered in the RT-qPCR analysis. 

### Protein expression analysis

To analyze if the changes observed in gene expression were translated into changes at protein level, we performed a Western blot experiment of the two most differentially expressed genes *SCN2B* and *KCNJ5*. We did not found statistically significant differences between the DCM group and the CNT group in the SCN2B protein levels (78 ± 19 *vs.* 100 ± 31, respectively), and the same results were obtained comparing these two groups in the KCNJ5 protein levels (128 ± 34 vs. 100 ± 27, respectively) (data not shown).

### Pathway characterization

To test whether the differentially expressed genes clustered into groups based on the biological process, or were related to one another, IPA was used. By applying the recommended parameters, we obtained a network that included the *SCN2B*, *KCNJ5*, *KCNJ8*, and *CACNB2* genes, and an additional network with the *CLIC2* gene.

**Figure 3:**
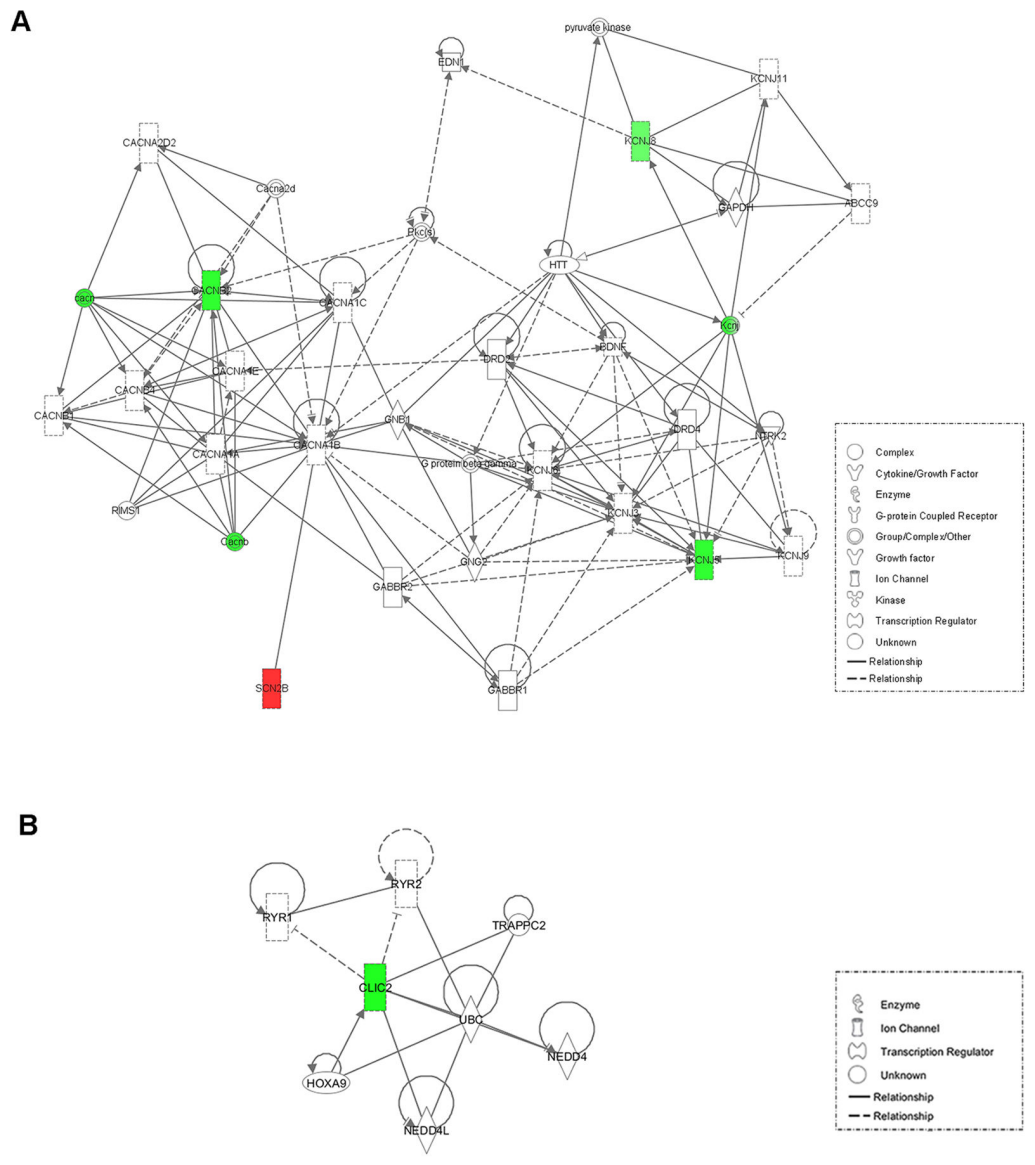
Functional network analysis of cardiac ion channel genes using IPA. **A**. First network including SCN2B, KCNJ5, KCNJ8 and CACNB2 genes (network score = 11). **B**. Second network with CLIC2 gene (network score = 3). Color intensity is correlated with fold change, green means downregulation and red overexpression. Straight lines indicate direct gene-to-gene interactions and dashed lines indirect interactions.

In the first network (Figure 3A), *CACNB2* showed direct interactions with gene families related to Ca^2+^ ion channels, such as the *CACN* and *CACNB* gene families. *RIM1*, a gene that regulates the voltage-gated calcium channels, is also associated with *CACNB2*. Finally, the sodium channel *SCN2B* is also closely related to *CACNB2* in this network through the Ca^2+^ channel *CACNA1B*. The potassium ion channel genes *KCNJ5* and *KCNJ8* interact with some of the 17 members of the inward rectifier K^+^ *KCNJ* family.

In the *CLIC2* network (Figure 3B), the inhibition of *RYR1* and *RYR2* (ryanodine receptor 1 and 2, respectively) was shown. In addition, *CLIC2* was related to *TRAPPC2* (trafficking protein particle complex 2). Finally, the ubiquitin system is related to *CLIC2* through *NEDD4* (E3 ubiquitin-protein ligase NEDD4), *NEDD4L* (E3 ubiquitin-protein ligase NEDD4-like), and *UBC* (ubiquitin C).

## Discussion

In the present study we carried out a microarray profiling of LV tissue from patients with DCM to investigate differential gene expression of ion channel genes in DCM compared to CNT group. Ordog et al. reported the gene expression of several ion channel subunits in healthy human cardiomyocytes, particularly comparing the ion channel gene expression between atrium and ventricle [4]. However, there have been no studies that have analyzed the expression level of genes related to these ion channels in human DCM and with a suitable sample size. Consequently, we focused on examining the expression levels of cardiac ion channels relevant to the contraction process.

Microarray experiments are a suitable method for analyzing the global expression of genes involved in human diseases, such as HF, showing alterations in gene expression profiles [23-26]. Moreover, there have been many studies that have used this method to examine expression levels of genes related to the EC process that occurs in muscle cells [27-29]. Besides, there are studies analyzing failing and non-failing human hearts establishing gender differences in electrophysiological gene expression, and using these data to predict the electrophysiological remodeling [30,31]. The DAVID gene functional classification tool allows sorting large gene lists into functionally related gene groups with an enrichment score, and summarizes the major biological importance of these gene groups. 

An alteration at the gene level in cardiac ion channels could produce an imbalance in the currents of the different ions involved in the contraction process of the cardiomyocyte. Since DCM is a disease resulting in the impairment of the cardiac contraction process [32,33], we aimed to study the alterations in the gene expression profile of 7 ion channels in DCM by comparing with control subjects and using a large sample size.

The results showed an upregulation of the *SCN2B* sodium channel and a downregulation of the potassium channels *KCNJ5* and *KCNJ8*, chloride channels *CLIC2* and *CLCN3*, and calcium channels *CACNA1C* and *CACNB2*. The differential mRNA expression levels of the genes *SCN2B*, *KCNJ5*, *KCNJ8*, *CLIC2*, and *CACNB2* were validated by RT-qPCR, while the calcium channel *CACNA1C* and the chloride channel *CLCN3* were not. The fact that these two genes could not be validated might be due to the high variability in DCM disease [34,35]. 

*SCN2B* was the only gene upregulated in DCM. However, when we measured the protein levels, we observed no significant changes in SCN2B protein. This sodium channel forms the β_2_-subunit of the voltage-gated, cardiac-specific sodium channel (*SCN5A*), which is responsible for the initiation of action potentials in the myocyte [12]. The α-subunit encoded by *SCN5A* forms the pore, and 2 auxiliary β-subunits (β_1_ and β_2_) modulate channel gating and cell surface expression levels and interact with the extracellular matrix and cell adhesion molecules. Moreover these auxiliary subunits play a key role in the regulation of the cardiac AP [36-37]. Therefore, differential expression of the α and β subunits can contribute to the ion flux alterations in HF. Our results show an upregulation of *SCN2B* gene, but a decreasing trend not significant compared to CNT in the level of SCN2B protein. This decreasing tendency has also been observed in other studies [38]. Possibly, this could be explained by control mechanisms such as post-translational modifications or degradation systems that occur in DCM patients. Furthermore, the absence of higher levels of SCN2B protein are consistent with the studies that show a reduction in the sodium current produced by the pore forming subunit SCN5A in HF [39]. 

Potassium channels *KCNJ5* and *KCNJ8* were downregulated in DCM, while the protein level of KCNJ5 remained unchanged. This observation could be explained by possible regulation mechanisms for the KCNJ5 protein. These mechanisms may be required to fine-tune levels of KCNJ5 activity and control its effect on cells, including potential post-transcriptional modifications. The *KCNJ5*-encoded protein Kir 3.4 can form both a homodimer and/or a heterodimer and is activated through various receptors coupled to G proteins modulating the channel complex opening [40]. Several studies confirm the importance of Kir3.4 to form a functional potassium channel [41]. The Kir 6.1 protein, encoded by *KCNJ8*, forms a heterodimer with the subunit Kir 6.2, establishing an entire pore complex [42]. In a *KCNJ8* knockout mouse, a progressive impairment in cardiac output was seen [43]. In analyzing the functions of these potassium channels, it seems that downregulating one or both of these in DCM patients could impair the current of K^+^ ions through the plasma membrane of the cardiomyocyte, provoking an alteration in the EC process, and consequently diminishing the ability of the heart to contract accordingly. 

The *CLIC2* gene encodes a protein belonging to the ubiquitous glutathione transferase structural family. These proteins are capable of transitioning from the aqueous phase into a phospholipid membrane, where they can function as ion channels [44]. There are studies that propose a regulatory role for *CLIC2* in the ryanodine receptor channel *RYR2* [45,46]. *RYR2* encodes a Ca^2+^ channel protein anchored to the sarcoplasmic reticulum (SR) in cardiomyocytes that triggers Ca^2+^ release to the cytoplasm during the contraction process. *CLIC2* acts as an inhibitor of RYR2 by binding directly and depressing Ca^2+^ release under resting conditions, thus favoring low cytoplasmic Ca^2+^ concentrations during diastole [45,47]. Takano et al. identified a mutation in *CLIC2* that resulted in abnormal cardiac function dependent on RYR channel activity [48]. This failure to inhibit RYR2, and thus increased cytoplasmic Ca^2+^ levels due to the downregulation of *CLIC2*, may alter the relaxation process in the myocyte and consequently explain the impaired EC process in DCM.

Our results showed a downregulation of the calcium channel *CACNB2*, while the expression of *CACNA1C* was not altered. The calcium channel encoded by the *CACNB2* gene is a membrane-associated guanylate kinase (MAGUK) protein that constitutes the β_2_ subunit of the L-type cardiac calcium channel *CACNA1C*. L-type calcium channels allow the influx of Ca^2+^ to the cytoplasm and are critical for controlling both cardiac excitability and EC coupling [11]. The pore forming subunit α contains the voltage sensor and is encoded by the *CACNA1C* gene, but its expression and functional properties are influenced by auxiliary subunits such as β_2_ [49,50]. Indeed, Yamaguchi et al. demonstrated that the β_2_ subunit increases the channel density and facilitates channel opening. This important role for the β_2_ subunit in the Ca^2+^ channel has been evidenced by its implication in several cardiovascular diseases such as short QT syndrome or Brugada syndrome [51,52]. Many groups have shown that mutations in genes encoding different β_2_ subunits and the pore forming α subunit are related to the pathology. The downregulation observed in the *CACNB2* gene may not properly inactivate the Ca^2+^ current through the *CACNA1C* α subunit, thereby altering the suitable cytoplasmic concentration of Ca^2+^ ions for the EC of cardiac muscle.

The gene expression profile of cardiac channels analyzed in this work has shown a general downregulation of all types of channels studied, with the exception of the sodium channel *SCN2B*, which is upregulated. This observation could explain the pathological process that occurs in DCM patients, where a general impairment of the contraction process may exist. As mentioned above, although no significant changes in its protein level have been found in our studies, the upregulation SCN2B gene could modify the sodim current. Therefore, it would be very interesting to address further protein expression studies to know if exists a regulation mechanism in these ion channel proteins related to clinical implications in DCM patients. The downregulation of *KCNJ5* and *KCNJ8* impairs the K^+^ current; and the downregulation of *CACNB2* and *CLIC2* leads to an increase in cytoplasmic Ca^2+^ ions, suggesting an altered time course for myocyte shortening and relaxation in DCM and a compromise in cardiac contractibility.

In addition, the sodium channel *SCN2B* is functionally coupled to *CACNB2*. It has been shown that a mutation in *CACNB2b*, another β subunit of the calcium channel, together with a mutation in *SCN5A*, underlies cardiac conduction disease [53]. Interestingly, there are other studies linking ion channels to each other through the observation of a complex interaction between the cardiac subunits of the sodium and transient potassium channels by coimmunoprecipitation experiments [54]. 

In the IPA analysis, we found two networks connecting different families of ion channels. In the first network, *CACNB2* showed interactions with the *CACN* gene family that comprises *CACNA1A*, *CACNA1C*, *CACNA1F*, *CACNA1S*, and *CACNB4*, which provides instructions for forming functional calcium channels [55,56]. *CACNB2* also interacts with the *CACNB* gene family (*CACNB* genes 1–4) that encodes MAGUK proteins and that function as auxiliary β subunits in the assembly and gating of voltage-gated Ca^2+^ channels [57]. Finally, *CACNB2* is associated with the regulator of the voltage-gated calcium channels *RIM1* [58], and with the sodium channel *SCN2B*, through its interaction with *CACNA1B*, since it has been described that a mutation in *CACNB2b*, and a mutation in *SCN5A* underlie cardiac conduction disease, as mentioned above [53].. The potassium ion channel genes *KCNJ5* and *KCNJ8* interact with some of the 17 members of the inward rectifier K^+^ *KCNJ* family, including *KCNJ3*, *KCNJ6*, *KCNJ9*, and *KCNJ11*. 

Another network revealed in the IPA analysis showed that *CLIC2* is an inhibitor of *RYR1* and *RYR2*, which function as calcium release channels in the SR by this chloride channel [59]. In addition, *CLIC2* is related to *TRAPPC2*, which is involved in the endoplasmic reticulum-to-Golgi transport vesicles [60]. Finally, the ubiquitin system is related to *CLIC2* through *NEDD4*, *NEDD4L*, and *UBC* (ubiquitin C), which regulate the interaction between the motor neurons and the muscle [61] or the current of ion channels [62-64].

All these data reveal a communication between cardiac ion channels, where a minimum alteration could produce a general injury in the normal function of the heart, affecting the EC coupling.

A common limitation of the studies that use cardiac tissues from end-stage failing human hearts is the fact that there is a high variability in disease etiology and treatment. To make our study population etiologically homogeneous, we chose DCM patients that did not report any family history of the disease. In addition, the patients used in this study were on conventional therapy and certain treatments may influence ion channel mRNA levels. Moreover, our tissue samples are confined to transmural left ventricle apex, so our findings could not be generalized to all layers and regions of the left ventricle, as well as to only cardiomyocytes. However our group has extensively used samples from human LV tissue and in techniques as electron microscopy it has been shown the presence of a high number of cardiomyocytes in these samples [65-68].

In conclusion, in our study we analyzed the gene expression of ion channels involved in cardiac muscle contraction in DCM patients compared with CNT group. The changed expression levels shown in the ion channel genes might partly underlie the altered shortening and relaxation process observed in this pathology. Our results may constitute the basis to modulate the contractibility impairment observed in DCM, associated with differential mRNA levels in ion channel genes.

## Supporting Information

Figure S1 **Gene expression of KCND3 and *ATPA2A* as positive controls of RT-qPCR experiments.** The graph shows the relative mRNA levels of RT-qPCR experiment normalized to the mRNA expression of 3 housekeeping genes (GAPDH, PGK1, and TFRC). The error bar represents the standard error of the mean (SEM) for DCM (n =21) and CNT (n = 8) samples in RT-qPCR data. * p < 0.05; ** p < 0.01.
(TIF)

Table S1 **Gene expression differences between DCM and CNT groups.** p < 0.01 and FC ≥ 1.3.
(XLSX)

Table S2 **Cardiac voltage-gated ion channel genes identified by DAVID programme based on the microarray results.**
(XLSX)
