# Supplementary figures and images for: Differential Gene Expression of Cardiac Ion Channels in Human Dilated Cardiomyopathy
Source: PLoS One. 2013 Dec 5;8(12):e79792. doi: 10.1371/journal.pone.0079792 (PMC3855055; doi:10.1371/journal.pone.0079792)

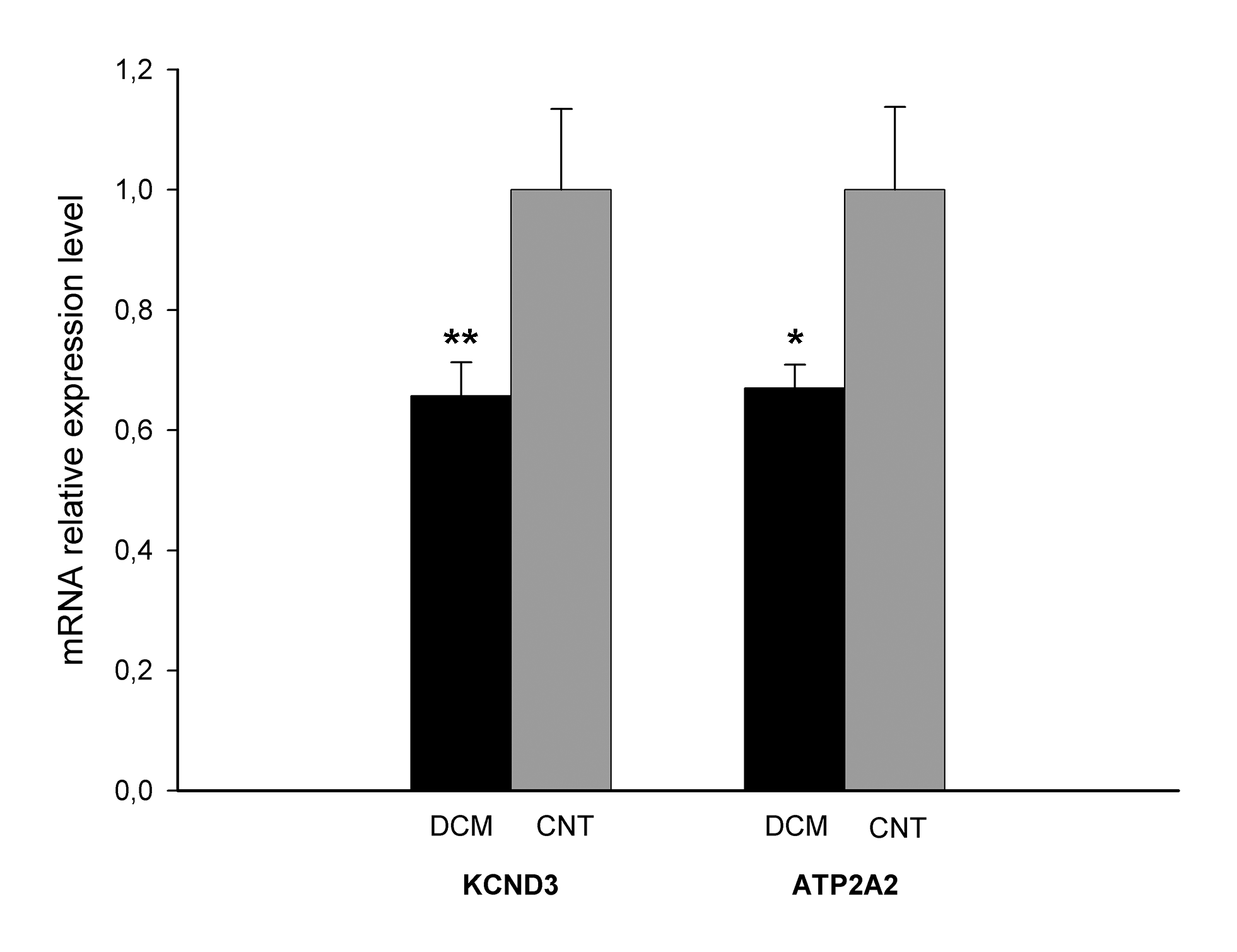

Supplement: Figure S1 — Gene expression of KCND3 and ATPA2A as positive controls of RT-qPCR experiments. The graph shows the relative mRNA levels of RT-qPCR experiment normalized to the mRNA expression of 3 housekeeping genes (GAPDH, PGK1, and TFRC). The error bar represents the standard error of the mean (SEM) for DCM (n =21) and CNT (n = 8) samples in RT-qPCR data. * p < 0.05; ** p < 0.01. (TIF) [file pone.0079792.s001.tif]
